# Vitamin D deficiency in Hashimoto’s thyroiditis: mechanisms, immune modulation, and therapeutic implications

**DOI:** 10.3389/fendo.2025.1576850

**Published:** 2025-08-01

**Authors:** Wencong Sun, Chao Ding, Yichen Wang, Guoqing Li, Zijie Su, Xinhui Wang

**Affiliations:** ^1^ Department of Thyroid Surgery, Henan Provincial People’s Hospital, People’s Hospital of Zhengzhou University, Zhengzhou, China; ^2^ Department of Geriatrics, Henan Provincial People’s Hospital, People’s Hospital of Zhengzhou University, Zhengzhou, China

**Keywords:** vitamin D, Hashimoto’s thyroiditis, thyroid function, antibody levels, autoimmunity

## Abstract

**Background:**

To evaluate vitamin D’s role in thyroid autoimmunity modulation, establish evidence-based supplementation protocols, and address surgical implications in Hashimoto’s thyroiditis (HT) care. Vitamin D deficiency is prevalent in HT patients and correlates with accelerated autoimmune progression. This review synthesizes mechanistic insights and clinical implications of vitamin D repletion in HT management.

**Objective:**

To evaluate vitamin D’s role in thyroid autoimmunity modulation, establish evidence-based supplementation protocols, and address surgical implications in HT care.

**Key Findings:**

Pathogenic Mechanism: Vitamin D deficiency (25(OH)D <20 ng/mL) disrupts VDR-mediated Treg/Th17 balance, increasing anti-TPO titers by 40–60% and hypothyroidism progression risk. Therapeutic Window: Supplementation (2000–4000 IU/day) reduces antibodies by 15–30% only in euthyroid TPOAb+ patients with baseline deficiency (<20 ng/mL), but efficacy diminishes in overt hypothyroidism. Surgical Imperative: Preoperative optimization (25(OH)D >30 ng/mL) lowers post-thyroidectomy hypocalcemia risk by 50% in HT patients.

**Conclusion:**

Vitamin D modulates HT through immune pathway regulation, yet response heterogeneity necessitates: Genotype-guided dosing (VDR-FokI FF carriers require 30% lower doses). Vitamin D supplementation has demonstrated potential to modulate immune responses, alleviate symptoms, and improve quality of life.

## 1 Introduction

Hashimoto’s thyroiditis (HT), an organ-specific autoimmune disorder characterized by lymphocytic infiltration and thyroid peroxidase (TPO) antibody production, has become the leading cause of hypothyroidism worldwide. Emerging evidence highlights vitamin D’s dual role in bone metabolism and immune regulation, particularly through its interaction with vitamin D receptors (VDR) expressed on immune cells (1, 2). This review systematically examines the tripartite relationship between vitamin D status, thyroid dysfunction, and autoimmune activity in HT, with particular focus on three key aspects: VDR polymorphisms in HT susceptibility; Vitamin D-mediated immunomodulatory mechanisms; Clinical implications of vitamin D repletion therapy. Vitamin D deficiency has been associated with various autoimmune diseases, including HT. Studies indicate that patients with HT often exhibit lower levels of vitamin D compared to healthy individuals, suggesting a potential link between vitamin D deficiency and the development of autoimmune thyroid disorders (3). The immunomodulatory properties of vitamin D are thought to be mediated through its interaction with the VDR, which is present in various immune cells (2). This interaction can influence the differentiation and function of T cells, promoting an anti-inflammatory environment and potentially reducing the autoimmune attack on thyroid tissue. For instance, vitamin D has been shown to inhibit the production of pro-inflammatory cytokines while enhancing the activity of regulatory T cells (4), which are crucial for maintaining immune tolerance (5).

Several clinical trials have investigated the effects of vitamin D supplementation on thyroid function and autoimmune markers in patients with HT. For example, a study reported that supplementation with cholecalciferol (vitamin D3) significantly reduced TPO antibody levels in vitamin D-deficient patients with HT, indicating an improvement in autoimmune activity (6). This reduction in antibody levels was accompanied by improvements in thyroid function, as evidenced by decreased thyroid-stimulating hormone (TSH) levels. The findings underscore the potential of vitamin D supplementation as a therapeutic strategy for managing autoimmune thyroid disorders, particularly in patients with concurrent vitamin D deficiency.

Moreover, the relationship between vitamin D levels and thyroid function appears to be complex. Some studies have reported a negative correlation between serum vitamin D levels and TSH, suggesting that lower vitamin D levels may be associated with higher TSH levels and, consequently, greater thyroid dysfunction (7). This correlation indicates that vitamin D may play a role in the progression of hypothyroidism in patients with HT. Additionally, the presence of vitamin D receptors in thyroid cells suggests that vitamin D may directly influence thyroid hormone production and secretion, further linking vitamin D status to thyroid health.

In conclusion, the current body of literature supports the hypothesis that vitamin D status significantly impacts thyroid function and autoimmune activity in HT patients. Given the immunomodulatory actions of vitamin D, supplementation may offer a viable adjunctive treatment for managing HT, particularly in individuals with vitamin D deficiency. Future research should focus on larger, randomized controlled trials to establish optimal dosing strategies and to further elucidate the mechanisms by which vitamin D influences thyroid autoimmunity. This could enhance our understanding of HT management and improve patient outcomes through targeted nutritional interventions.

### 1.1 Epidemiological status of HT

#### 1.1.1 Incidence and risk factors of HT

HT is the most prevalent autoimmune disorder affecting the thyroid gland, characterized by the destruction of thyroid cells due to lymphocytic infiltration and the presence of autoantibodies. The incidence of HT varies globally, with higher rates observed in women, particularly in those with a family history of autoimmune diseases. Several risk factors have been identified, including genetic predisposition, environmental triggers, and nutritional deficiencies. Vitamin D deficiency, for instance, has been linked to an increased risk of developing HT, as it plays a crucial role in immune regulation. Studies have shown that individuals with lower serum levels of 25-hydroxyvitamin D are more likely to test positive for thyroid peroxidase antibodies, a hallmark of HT (8). Furthermore, the presence of other autoimmune conditions, such as celiac disease and type 1 diabetes, can also elevate the risk of HT, suggesting a shared pathogenic mechanism among autoimmune disorders (9). This multifactorial etiology indicates that both genetic and environmental factors contribute to the development of HT, necessitating a comprehensive approach to prevention and management.

#### 1.1.2 Prevalence of vitamin D deficiency in patients with HT

Vitamin D deficiency is notably prevalent among patients with HT, with numerous studies highlighting its association with the disease. Research indicates that patients with HT often exhibit significantly lower levels of serum 25-hydroxyvitamin D compared to healthy controls (10). This deficiency may exacerbate autoimmune processes, as vitamin D is known for its immunomodulatory properties, helping to maintain immune tolerance and reduce inflammation. A systematic review has shown that vitamin D supplementation can lead to a decrease in thyroid autoantibody levels, particularly thyroid peroxidase antibodies, in patients with HT (11). Additionally, vitamin D status has been correlated with thyroid function, where lower levels of vitamin D are associated with higher TSH levels, indicating a potential link between vitamin D deficiency and hypothyroidism (12). The complex interplay between vitamin D and thyroid autoimmunity suggests that addressing vitamin D deficiency may be a crucial component in the management of HT, potentially improving clinical outcomes and quality of life for affected individuals. Further research is necessary to establish optimal vitamin D levels and the efficacy of supplementation in this population.

### 1.2 The biological functions and mechanisms of vitamin D

#### 1.2.1 Synthesis and metabolism of vitamin D

Vitamin D synthesis initiates when skin ultraviolet radiation b (UVB) exposure converts 7-dehydrocholesterol to previtamin D3, which thermally transforms into vitamin D3 (cholecalciferol). The liver hydroxylates it to 25-hydroxyvitamin D [25(OH)D], the circulatory biomarker, followed by renal conversion to active 1,25-dihydroxyvitamin D [1,25(OH)2D]. This hormonal form binds VDR, regulating calcium homeostasis, immune responses, and musculoskeletal functions. Synthesis efficiency depends on age (declining with aging), skin melanin content (darker skin reduces UVB absorption), latitude, and diet (fatty fish, fortified foods, supplements) (13). Limited sun exposure necessitates dietary supplementation. Vitamin D deficiency correlates with osteoporosis, autoimmune disorders, and infection susceptibility, underscoring its clinical relevance in disease prevention and management (14).

#### 1.2.2 Vitamin D receptor and its signaling pathways

The VDR, a nuclear receptor superfamily member expressed in intestines, kidneys, and immune cells, binds 1,25(OH)2D to regulate gene expression via vitamin D response elements (VDREs). Beyond mediating calcium/phosphate homeostasis and cell differentiation, VDR signaling intersects with inflammatory pathways through molecular crosstalk. For example, VDR inhibits NF-κB nuclear translocation by stabilizing IκBα (15), while MAPK pathway modulation involves VDR-dependent suppression of p38 phosphorylation in macrophages (16). Recent studies highlight tissue-specific immune regulation: in HT, thyroid-resident regulatory T cells (tTregs) exhibit upregulated VDR expression, and calcitriol enhances their suppressive capacity against thyroglobulin antibody production (17). Epigenetically, hypermethylation of the VDR promoter in CD4+ T cells correlates with reduced VDR expression and aggravated thyroid autoimmunity (18). Vitamin D also primes dendritic cells through histone H3K27 acetylation at VDRE loci, enhancing tolerogenic phenotypes. These discoveries clarify mechanistic gaps and reveal therapeutic opportunities, such as combining vitamin D analogs with demethylating agents to restore VDR signaling in autoimmune diseases.

#### 1.2.3 The role of vitamin D in the immune system

**Figure 1:**
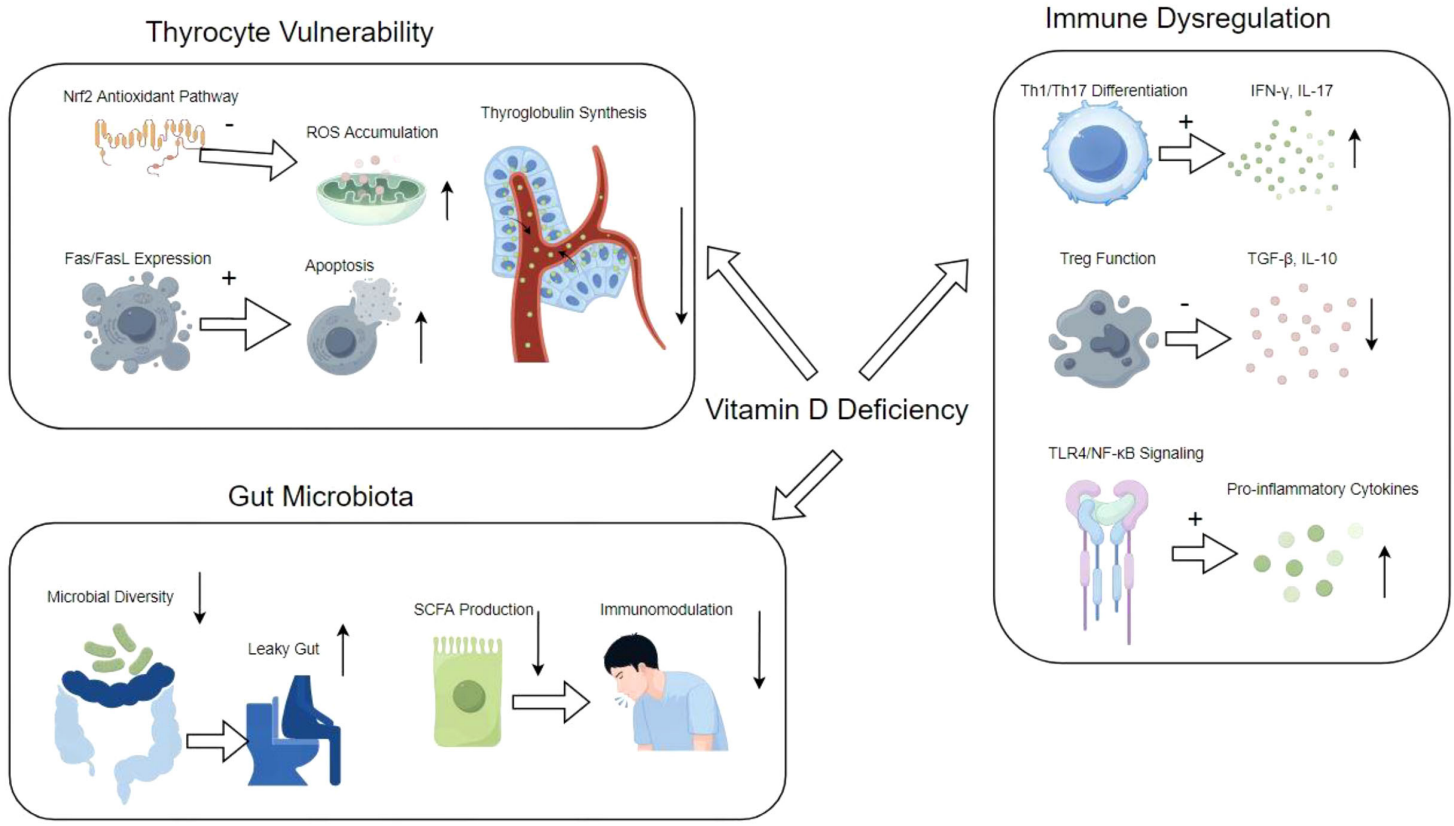
Vitamin D Deficiency in Hashimoto’s Thyroiditis Integrated Mechanisms.

Vitamin D plays a significant role in the immune system by modulating both innate and adaptive immune responses. It enhances the pathogen-fighting effects of monocytes and macrophages, promoting the production of antimicrobial peptides such as cathelicidin and defensins. Additionally, vitamin D influences the differentiation and function of T cells, particularly by suppressing the activation of pro-inflammatory Th1 and Th17 cells while promoting regulatory T cells that help maintain immune tolerance. This immunomodulatory effect is particularly relevant in the context of autoimmune diseases, where vitamin D deficiency has been associated with increased disease activity and severity. For example, in HT, lower levels of vitamin D correlate with higher levels of thyroid autoantibodies and thyroid dysfunction. Supplementation with vitamin D has been shown to reduce these autoantibody levels and improve clinical outcomes in patients with autoimmune thyroid diseases. Furthermore, vitamin D’s role in enhancing the gut barrier function and modulating the gut microbiome also contributes to its protective effects against inflammatory diseases. The comprehensive mechanism of HT, and vitamin D deficiency is illustrated in **Figure 1**. Overall, adequate vitamin D levels are crucial for optimal immune function and may play a preventive role in various autoimmune conditions and infections (7, 19, 20).

### 1.3 The relationship between vitamin D status and thyroid function

#### 1.3.1 The impact of vitamin D deficiency on thyroid hormone synthesis

Vitamin D deficiency has been increasingly recognized as a significant factor influencing thyroid hormone synthesis, particularly in autoimmune thyroid diseases such as HT. Research indicates that vitamin D plays a crucial role in the regulation of the immune system, which is essential for maintaining thyroid health. In patients with HT, low levels of vitamin D have been correlated with increased levels of thyroid peroxidase antibodies (anti-TPO), which are indicative of thyroid autoimmunity (21). Furthermore, vitamin D is believed to exert immunomodulatory effects that can mitigate the inflammatory processes involved in autoimmune conditions, potentially reducing the destruction of thyroid tissue and the subsequent decline in hormone production. Studies have shown that supplementation with vitamin D can lead to a significant decrease in anti-TPO antibody levels and an improvement in thyroid function parameters (6). This suggests that vitamin D may enhance thyroid hormone synthesis by modulating immune responses and reducing the autoimmune attack on thyroid cells, thus highlighting the importance of maintaining adequate vitamin D levels for optimal thyroid health.

#### 1.3.2 The correlation between vitamin D and hypothyroidism

The correlation between vitamin D levels and hypothyroidism has been the subject of extensive investigation, particularly in the context of autoimmune thyroid diseases. Numerous studies have reported that individuals with hypothyroidism, especially those with HT, often exhibit significantly lower serum levels of vitamin D compared to healthy controls (22). A meta-analysis has confirmed that vitamin D deficiency is prevalent among patients with autoimmune thyroid diseases, with a notable association between low vitamin D levels and increased TSH levels, suggesting a potential link to the development of hypothyroidism (23). Moreover, vitamin D has been shown to have a protective role against the progression of thyroid dysfunction, as evidenced by findings that higher vitamin D levels are associated with lower odds of developing hypothyroidism (24). This relationship underscores the potential for vitamin D supplementation as a therapeutic strategy in managing thyroid disorders, particularly in patients presenting with vitamin D deficiency. However, further research is necessary to elucidate the mechanisms underlying this association and to establish definitive clinical guidelines regarding vitamin D supplementation in hypothyroid patients.

### 1.4 The impact of vitamin D on antibody levels

#### 1.4.1 The relationship between vitamin D and thyroid antibodies

Vitamin D plays a significant role in the pathogenesis of autoimmune diseases such as HT. Numerous studies have indicated a higher prevalence of vitamin D deficiency in patients with HT compared to healthy individuals. Specifically, research has shown a negative correlation between serum levels of 25-hydroxyvitamin D (25(OH)D) and the levels of thyroid peroxidase antibodies (anti-TPO) and thyroglobulin antibodies (anti-Tg) in these patients. For instance, one study highlighted that lower vitamin D levels were associated with elevated anti-TPO antibody titers, suggesting that vitamin D deficiency may exacerbate autoimmune responses in HT patients (20). Furthermore, vitamin D’s immunomodulatory actions are thought to contribute to its role in reducing thyroid autoantibody levels, potentially by inhibiting pro-inflammatory cytokines and promoting immune tolerance (25). This relationship underscores the importance of maintaining adequate vitamin D levels to potentially mitigate the autoimmune processes involved in thyroid disorders.

#### 1.4.2 The regulatory effect of vitamin D supplementation on antibody levels

Vitamin D supplementation has been shown to have a beneficial effect on antibody levels in patients with autoimmune thyroid diseases, including HT. Clinical trials have demonstrated that vitamin D can significantly reduce the levels of thyroid antibodies, particularly anti-TPO and anti-Tg antibodies. For example, a meta-analysis of randomized controlled trials revealed that vitamin D supplementation led to a significant decrease in TPO antibody titers, suggesting a direct impact on thyroid autoimmunity (26). Additionally, a study indicated that patients receiving vitamin D supplementation exhibited a 30.5% reduction in anti-TPO antibody levels compared to a 16.5% reduction in the placebo group, highlighting the efficacy of vitamin D in modulating autoimmune responses (6). Moreover, prolonged supplementation (greater than three months) was associated with more pronounced reductions in antibody levels and improvements in thyroid function, suggesting that maintaining adequate vitamin D levels could be an important therapeutic strategy for managing autoimmune thyroid conditions (27). These findings collectively advocate for the incorporation of vitamin D supplementation in treatment regimens for patients with autoimmune thyroid diseases to enhance their clinical outcomes and reduce antibody-mediated damage to the thyroid gland.

### 1.5 The potential impact of vitamin D supplementation on patient prognosis

#### 1.5.1 Evaluation of clinical studies and supplementation effects

Clinical research has increasingly focused on the role of vitamin D in modulating immune responses and its potential therapeutic effects in various autoimmune diseases, including HT. Numerous studies have demonstrated a significant correlation between low vitamin D levels and the prevalence of HT, suggesting that vitamin D deficiency may contribute to the pathogenesis of this autoimmune disorder (5). For instance, a prospective study indicated that vitamin D supplementation led to a notable decrease in thyroid autoantibody titers among patients with HT, highlighting its immunomodulatory properties (6). Furthermore, vitamin D’s role in enhancing immune tolerance is particularly relevant in autoimmune conditions, where it may help to mitigate the autoimmune attack on thyroid tissues (28).

The evidence supporting vitamin D’s efficacy in improving clinical outcomes is compelling. A systematic review found that vitamin D supplementation was associated with reduced thyroid peroxidase antibody levels, which are critical markers of autoimmune thyroid disease (29). Additionally, a meta-analysis confirmed that patients with HT exhibited significantly lower serum levels of 25-hydroxyvitamin D compared to healthy controls, reinforcing the notion that vitamin D deficiency may exacerbate autoimmune processes (30). Despite these promising findings, the literature also underscores the need for further randomized controlled trials to establish optimal dosing regimens and long-term effects of vitamin D supplementation on thyroid autoimmunity and overall patient prognosis (6). Over the past decade, numerous investigations have indicated a notable decrease in TPOAb and/or TgAb levels among adult individuals diagnosed with autoimmune thyroiditis following vitamin D supplementation across diverse populations (**Table 1**). The dosages administered for vitamin D supplementation have varied considerably, ranging from 1000 to 4000 IU per day for periods of 1 to 6 months, or weekly doses of 50,000 to 60,000 IU for durations spanning 2 to 36 months. This extensive variation in both the dosage and the length of vitamin D supplementation renders it challenging to define and subsequently recommend an ideal vitamin D protocol for patients with HT.

**Table 1:** Multiple studies showing a significant reduction in antithyroid antibody levels following vitamin D supplementation.

Author and Year	Number of participants (F/M)	Dose of supplementation duration	Effect
Chahardoli et al, 2019 (31)	42 F	50,000 IU weekly, 8 months	It decreased anti-TG Ab and TSH, but had no significant effect on anti-TPO Ab and T3/T4
Vahabi Anaraki et al., 2017 (32)	36 F/20 M	50,000 IU weekly, 12weeks	Serum 25(OH)D level was significantly increased, while anti-TPO antibody was not significantly decreased
Krysiak et al., 2019 (33)	36 M	4,000 IU daily, 26 weeks	The levels of anti-TPO and anti-TG antibodies in the treatment group were significantly lower than those in the control group
Krysiak et al., 2017 (34)	34 F	2000 IU daily, 6 months	Both TPOAb and TgAb decreased, and the decrease in TPOAb was more significant than that in TgAb
Knutsen et al., 2017 (35)	181 F/69 M	1000 IU daily, 16weeks	There were no significant changes in TPOAb and TSH
Villa et al., 2020 (36)	180 F/18 M	100,000 IU/month, 3 months-3years	TSH significantly decreased, while TPOAb and TgAb were not detected
Nodehi et al., 2019 (37)	48 F	50,000 IU weekly, 3 months	The Th17/Tr1 ratio was significantly decreased, and TPOAb and TgAb were not detected
Simsek et al., 2016 (38)	68 F/14 M	1000 IU daily, 1 month	TPO-Ab and TgAb levels were significantly decreased
Krysiak et al., 2021 (39)	67M	100μg daily, 6 months	The TPOAb level has decreased significantly
Krysiak et al., 2022 (40)	62 F	4000 IU daily, 6 months	The decrease of antibody titer in the experimental group was greater than that in the control group
Mazokopakis et al., 2015 (41)	180 F/38 M	1200–4000 IU daily, 4 months	The anti-TPO level decreased significantly, while there was no statistical significance in anti-TG and TSH
Chaudhary et al., 2016 (42)	76 F/24 M	60,000 IU weekly, 8weeks	The median reduction of TPO-Ab in the intervention group was significantly higher than that in the control group, and the reduction of TPO-Ab in the intervention group was more significant in patients with TSH ≤ 10 mIU/L

#### 1.5.2 Development and implementation of vitamin D supplementation protocols

The establishment of effective vitamin D supplementation protocols is crucial for enhancing patient outcomes, particularly in populations at risk of deficiency, such as those with autoimmune thyroid disorders. Given the high prevalence of vitamin D deficiency in patients with HT, healthcare providers are encouraged to adopt systematic approaches for screening and supplementation (43). This may include routine assessment of serum vitamin D levels in patients diagnosed with HT, followed by individualized supplementation strategies based on the severity of deficiency.

For example, a study involving patients with HT demonstrated that administering cholecalciferol (vitamin D3) at a dose of 60,000 IU weekly for eight weeks resulted in a significant reduction in anti-thyroid antibody levels, suggesting that structured supplementation can effectively modulate immune responses (6). Moreover, implementing educational initiatives for both healthcare providers and patients about the importance of maintaining adequate vitamin D levels can further improve adherence to supplementation protocols (28).

In practice, multidisciplinary collaboration among endocrinologists, dietitians, and primary care physicians is essential to ensure comprehensive care. This collaboration can facilitate the integration of dietary recommendations alongside supplementation, promoting a holistic approach to managing autoimmune thyroid diseases (5). Ultimately, the successful implementation of vitamin D supplementation protocols has the potential to significantly enhance the clinical prognosis of patients with HT and other related autoimmune disorders.

## 2 Discussion

The interplay between vitamin D status and HT has generated substantial interest, yet it remains a field marked by both consensus and controversy. This section synthesizes divergent perspectives, unresolved questions, and emerging paradigms while addressing critical research gaps and future directions.

### 2.1 Divergent perspectives and controversies

#### 2.1.1 The immunomodulatory role of vitamin D: mechanism *vs*. association

While observational studies consistently report an inverse correlation between serum vitamin D levels and thyroid autoantibodies (e.g., anti-TPO and anti-Tg), the causal relationship remains contentious. Proponents of vitamin D’s therapeutic potential argue that its immunomodulatory properties—mediated via VDR signaling—directly suppress autoimmune activity by promoting regulatory T-cell (Treg) differentiation and inhibiting pro-inflammatory Th17 responses (34). However, skeptics emphasize that these findings may reflect epiphenomenal associations rather than causality. For instance, vitamin D deficiency could result from chronic inflammation in HT rather than driving autoimmunity itself. Randomized controlled trials (RCTs) have yielded mixed results: while some demonstrate significant reductions in antibody titers post-supplementation (34), others report minimal clinical impact (44). Notably, recent studies reveal critical limitations: a double-blind RCT (n=150) found no improvement in thyroid antibody levels despite normalizing serum 25(OH)D in HT patients while another trial observed transient antibody reduction but no sustained benefit after 12 months (6, 11, 27, 45). This discrepancy may stem from heterogeneity in baseline vitamin D levels, dosing regimens, or genetic polymorphisms in VDR genes, which modulate individual responsiveness to supplementation. Specifically, retrospective analyses suggest vitamin D supplementation ≥2000 IU/day may suppress autoantibodies only in subgroups with baseline 25(OH)D <20 ng/mL and specific VDR-FokI genotypes, highlighting the limitations of universal dosing recommendations (46).

#### 2.1.2 Dose-response dynamics: optimal supplementation strategies

The optimal dosage and duration of vitamin D supplementation in HT remain unresolved. Some researchers advocate aggressive repletion (e.g., 60,000 IU/week for 8 weeks) to rapidly normalize serum 25(OH)D levels (34), whereas others caution against potential toxicity and favor conservative dosing (e.g., 1,000–4,000 IU/day). A meta-analysis by Zhang et al. (2021) found no linear dose-response relationship between vitamin D intake and antibody reduction, suggesting threshold effects beyond which additional benefits plateau (26). Furthermore, the interplay between vitamin D and other nutrients, such as selenium—a cofactor for thyroid hormone synthesis—complicates clinical recommendations. Trials combining vitamin D with selenium have shown synergistic effects on antibody reduction, yet the mechanisms remain unclear (47).

### 2.2 Fundamental questions and unresolved issues

#### 2.2.1 Temporal dynamics: Does early intervention alter disease trajectory?

A critical gap lies in understanding whether vitamin D supplementation during subclinical or early-stage HT can delay progression to overt hypothyroidism. Longitudinal studies are sparse, but preliminary data suggest that early repletion may stabilize thyroid function in euthyroid patients. Conversely, trials in advanced HT often fail to reverse established damage, highlighting the need for preventive strategies.

#### 2.2.2 Genetic and epigenetic influences

Emerging evidence implicates VDR gene polymorphisms (e.g., FokI and BsmI) in modulating HT susceptibility and vitamin D responsiveness (48). For example, carriers of the FokI FF genotype exhibit enhanced VDR transcriptional activity and greater antibody reduction post-supplementation (49). However, these findings are not universally replicated, underscoring the complexity of gene-environment interactions. Epigenetic modifications, such as DNA methylation of VDR promoters in immune cells, may further explain interindividual variability.

#### 2.2.3 Beyond antibodies: systemic and tissue-specific effects

While most studies focus on antibody titers and TSH levels, the systemic impact of vitamin D on thyroid histopathology and extrathyroidal manifestations (e.g., fatigue, cognitive dysfunction) is poorly characterized. Animal models reveal that vitamin D deficiency exacerbates lymphocytic infiltration and follicular destruction in the thyroid (50), yet human histopathological data are lacking. Additionally, vitamin D’s pleiotropic effects on gut microbiota and barrier integrity—factors implicated in autoimmune pathogenesis—warrant exploration in HT.

#### 2.2.4 Surgical implications: vitamin D in perioperative thyroid care

The role of vitamin D in thyroidectomy outcomes remains highly controversial. While some propose correcting deficiency to reduce postoperative complications (e.g., hypocalcemia, RLN recovery), surgical cohort studies refute this association:

In thyroid lobectomy cohorts (n=365), preoperative vitamin D deficiency (25(OH)D <20 ng/mL) did not correlate with recurrent laryngeal nerve paralysis rates (2.5% *vs*. 2.8% in sufficient group, P=0.82) or recovery time (51).

Similarly, vitamin D supplementation ≥2000 IU/day failed to reduce transient hypocalcemia risk (52), contrasting with selenium’s established protective effects.

These findings suggest that: (1) HT-related surgical risks may be immune-mediated rather than nutrient-dependent; (2) Vitamin D’s immunomodulation might require longer intervention windows (>6 months) to impact surgical outcomes. Future trials should stratify by thyroid autoimmunity severity and surgical urgency.

### 2.3 Research gaps and future directions

#### 2.3.1 Mechanistic studies: bridging molecular insights to clinical practice

Current evidence relies heavily on observational correlations, necessitating mechanistic studies to elucidate how vitamin D modulates thyroid-specific immune responses. Techniques such as single-cell RNA sequencing of thyroid-infiltrating lymphocytes and VDR knockout models could clarify its direct *vs*. indirect effects. Furthermore, the role of local vitamin D metabolism within thyroid tissue—mediated by enzymes like CYP27B1—remains unexplored (53). Furthermore, vitamin D’s local effects on thyroid surgical wound healing and nerve regeneration merit investigation using RLN injury models (54).

#### 2.3.2 Personalized medicine: biomarker-driven supplementation

Future RCTs should stratify participants by baseline vitamin D status, VDR genotypes, and autoimmune phenotypes to identify subgroups most likely to benefit. Biomarkers such as serum cathelicidin (LL-37)—a vitamin D-dependent antimicrobial peptide—may predict therapeutic responses (55).

#### 2.3.3 Long-term outcomes and combination therapies

No studies have evaluated the decade-scale impact of vitamin D supplementation on thyroid volume, nodularity, or cancer risk in HT. Additionally, combining vitamin D with immunomodulators (e.g., low-dose naltrexone) or thyroid hormone analogs could enhance efficacy. A pilot study of calcitriol (1,25(OH)2D3) plus levothyroxine showed superior antibody reduction compared to monotherapy (56), but larger trials are needed.

#### 2.3.4 Global health perspectives: addressing disparities

Geographic disparities in vitamin D status among HT patients reveal critical intersections of latitude-driven sunlight exposure, socioeconomic factors, and public health policies. While Nordic countries (e.g., Sweden: 55-69°N) recommend daily 20 μg vitamin D supplementation year-round for adults – a policy correlating with 20% higher population 25(OH)D levels since 2018 (57, 58). Mediterranean nations like Italy (35-47°N) lack mandatory fortification programs, resulting in 62% of HT patients having <50 nmol/L 25(OH)D (59). Conversely, India (8-37°N), despite abundant sunlight, it was shown that 48% of HT patients had high peroxidase antibodies and that the degree of vitamin D deficiency was associated with a vegetarian-related inadequate diet and unregulated fortification (60). Notably, Canada’s nationwide milk/plant-based milk fortification (≥1 μg/100 mL) has narrowed the 25(OH)D gap between HT and healthy cohorts by 34% (61). These contrasts underscore the need for latitude-adjusted guidelines: the 40th parallel (e.g., Beijing 39°N *vs*. Boston 42°N) may serve as a threshold for initiating targeted food fortification. Integrating such region-specific strategies with socioeconomic interventions (e.g., subsidized supplements in low-income regions) could reduce global HT morbidity disparities (62).

## 3 Conclusion

### 3.1 What we know

The relationship between vitamin D deficiency and HT is well-established, with robust evidence indicating that inadequate vitamin D levels exacerbate immune dysregulation, impair thyroid function, and elevate antibody levels in patients. Genetic, environmental, and immunological factors interact in this autoimmune condition, and vitamin D supplementation has demonstrated potential to modulate immune responses, alleviate symptoms, and improve quality of life. This evidence supports the role of vitamin D as a therapeutic adjunct in holistic management strategies.

### 3.2 What remains unclear

Key uncertainties persist regarding the precise molecular mechanisms through which vitamin D influences the pathophysiology of HT. Patient responses to supplementation are heterogeneous, likely due to variability in genetic predispositions, environmental triggers, and baseline vitamin D status. Furthermore, inconsistent study designs and differing methodologies for assessing vitamin D levels and thyroid function limit the generalizability of findings, complicating clinical interpretation and application.

### 3.3 What needs to be done

Future research must prioritize large-scale, RCTs vitamin D supplementation guidelines, optimize dosing regimens, and validate biomarkers for monitoring therapeutic efficacy. Interdisciplinary collaboration among endocrinologists, immunologists, and nutritionists is essential to unravel the complex interplay of factors driving disease progression. Additionally, mechanistic studies should focus on elucidating vitamin D’s immunomodulatory pathways to refine personalized treatment approaches and translate insights into evidence-based clinical practices that enhance patient outcomes.
